# Device-based Therapy for Hypertension

**DOI:** 10.1007/s11906-016-0670-5

**Published:** 2016-07-01

**Authors:** Fu L. Ng, Manish Saxena, Felix Mahfoud, Atul Pathak, Melvin D. Lobo

**Affiliations:** 1 Barts BP Centre of Excellence, Barts Heart Centre, St Bartholomew’s Hospital, W Smithfield, London, EC1A 7BE UK; 2 Barts NIHR Cardiovascular Biomedical Research Unit, Charterhouse Square, William Harvey Research Institute, Queen Mary University London, London, EC1M 6BQ UK; 3 Department of Internal Medicine, Cardiology, Angiology, Intensive Care Medicine, Saarland University Hospital, Homburg/Saar, Germany; 4 Department of Cardiovascular Medicine, Hypertension and Heart Failure Unit, Health Innovation Lab (Hi-Lab) Clinique Pasteur, Toulouse, France

**Keywords:** Hypertension, Renal denervation, Baroreflex activation, Carotid sinus stimulation, Arteriovenous anastomosis, Interventional devices, Coupler

## Abstract

Hypertension continues to be a major contributor to global morbidity and mortality, fuelled by an abundance of patients with uncontrolled blood pressure despite the multitude of pharmacological options available. This may occur as a consequence of true resistant hypertension, through an inability to tolerate current pharmacological therapies, or non-adherence to antihypertensive medication. In recent years, there has been a rapid expansion of device-based therapies proposed as novel non-pharmacological approaches to treating resistant hypertension. In this review, we discuss seven novel devices—renal nerve denervation, baroreflex activation therapy, carotid body ablation, central iliac arteriovenous anastomosis, deep brain stimulation, median nerve stimulation, and vagal nerve stimulation. We highlight how the devices differ, the varying degrees of evidence available to date and upcoming trials. This review also considers the possible factors that may enable appropriate device selection for different hypertension phenotypes.

## Introduction

Resistant hypertension (RHTN) continues to significantly contribute to the overall population’s cardiovascular morbidity and mortality [1]. Depending on the cohort assessed, the prevalence of RHTN is reported to range between 8 and 18 % [1, 2, 3•]. There are many aetiologies to RHTN, and the role of non-adherence to medications should not be understated [4]. This is often attributed to adverse drug effects, lifelong treatment for an asymptomatic disease and patient factors such as difficulty accommodating medications into activities of daily living. As a result, up to 50 % of newly diagnosed hypertensives are no longer taking their antihypertensive medications 6 months following initial prescription, and up to one quarter of patients with RHTN are not taking any of their antihypertensive drugs [5, 6].

Whilst many patients seem to express a preference for non-drug approaches to managing long-term conditions, historically the options for interventional treatments have been limited. However, the recent past has seen the introduction of a burgeoning array of device-based therapies for hypertension offering a more targeted approach to blood pressure (BP) lowering. In this review, we discuss seven novel device therapies, all at different stages of development and with varying degrees of evidence available to date. We highlight how they differ and consider potential factors that may enable appropriate device selection for different hypertension phenotypes.

## Renal Denervation

### Mechanism of Action

Of the novel device-based therapies for hypertension, renal denervation (RDN) has accumulated the largest body of evidence thus far. Current endovascular catheter systems typically access the renal arteries via the femoral artery and deliver radiofrequency (RF) or ultrasound (US) energy resulting in focal frictional heating of the arterial wall [7]. Other devices use neurotoxic agents such as alcohol or guanethedine [8]. This causes the destruction of the peri-arterial adventitial afferent and efferent renal nerves. The loss of sympathetic efferent nerve signalling may lead to decreased renin secretion by the juxtaglomerular apparatus, renal vasodilatation and sodium excretion [9]. Furthermore, removal of renal afferent nerve activity could also reduce sympathetic outflow from the central nervous system [10]. As the energy is delivered in an indiscriminate manner, including to the structures adjacent to the target nerves, damage to the endothelium and the tunica media has been observed in a porcine model [11]. It is this collateral damage that may contribute to adverse events as discussed below.

### Current Evidence

The first proof-of-concept open-label study, SYMPLICITY HTN-1, demonstrated that unipolar RF RDN was associated with a mean reduction in office BP of 22/10 mmHg [12•]. Subsequently, SYMPLICITY HTN-2 was a randomised open-labelled study, with the control group maintained on previous medical therapy for the first 6 months before being offered delayed RDN. There was a marked difference between the two groups at 6 months, with better office BP control by 33/11 mmHg in favour of RDN [13]. However, the fall in ambulatory BP levels was less impressive (11/7 mmHg) with the caveat that these measurements were available in only half the patients. Nonetheless, the beneficial impact appeared long-lasting, with a fall of 33/14 mmHg observed at 36 months post-procedure [14]. Over the 3-year follow-up period, there were only a small number of reported procedural complications, including one haematoma and one renal artery dissection arising from 52 interventions.

Up to 2014, studies of RDN have all been open-label, and thus criticised over the fact that a substantial placebo effect of device therapy may have arisen, particularly due to the invasive nature of the therapy which is associated with patient discomfort [15]. In early 2014, the first randomised sham-controlled trial of RDN, SYMPLICITY HTN-3, reported results that were less promising than the open-labelled trials. At 6 months, those who received RDN showed a reduction in office BP of 14/7 mmHg, which was comparable to the 12/5 mmHg drop in the sham (renal angiography only) group. There was also a lack of difference between the groups for ambulatory BP recordings [16••]. Whilst this was a disappointing finding for proponents of RDN, potential confounders that have been put forward to account for the negative results include the large proportion (40 %) of participants in both arms undergoing medication changes, and the surprising finding that only a small fraction (5 %) of patients undergoing RDN received per-protocol bilateral circumferential renal nerve ablation. When considering those that did receive per-protocol treatment, this subgroup exhibited the greatest reductions in office, home and ambulatory systolic BP (−24, −9, and −10 mmHg, respectively) but not reaching statistical significance in this subgroup post hoc analysis [17].

The importance of specialist centres involved in both selecting patients for RDN and delivering the therapy itself may be highlighted in the DENERHTN trial. This open-label randomised controlled trial recruited from 15 French specialist tertiary hypertension care centres, demonstrated that in a well-selected cohort, RDN in addition to a standardised stepped-care antihypertensive treatment (SSAHT) resulted in a SBP that was 5.9 mmHg lower than that in the control SSAHT group [18].

Although the sham-controlled clinical trial data has not been the most encouraging, registry data has been more heartening. The prospective Global SYMPLICITY Registry showed that in 998 patients, RDN resulted in a reduction in office and ambulatory systolic BP of 11.6 and 6.6 mmHg, respectively, at 6 months [19]. The single-centre ALSTER [20] and Heidelberg [21] registries also showed response rates of 76 and 73 %, as defined by reductions of systolic blood pressure ≥10 mmHg, respectively. The most recent collection of RDN experience within the UK recently identified 253 patients across 18 specialist hypertension centres where there was a mean fall in office and ambulatory BP of 22/9 and 12/7 mmHg, respectively [22]. Unsurprisingly, the fall in BP was the greatest in the highest quartile for baseline ambulatory systolic BP (mean 199 mmHg), where the decline in office and ambulatory BP was 30/12 and 22/13 mmHg, respectively. Although there were multiple medications changes, more antihypertensive drugs stopped (0.91/patient) as compared to started (0.36/patient), with similar numbers where doses were up-titrated (0.21/patient) and down-titrated (0.17/patient). Taken together, these real-world datasets support the efficacy of RDN and suggest the need for additional trial data.

The clinical trials to date demonstrate that RDN is a relatively safe procedure with a major adverse event rate of about 1.4 % [16••]. However, it has been demonstrated using optical coherence tomography that radiofrequency denervation may result in diffuse arterial constriction, oedema and thrombus formation which could form a substrate for future renovascular disorder [23]. On the other hand, both balloon-based and non-balloon-based technologies result in different patterns of thermal injury, including dissection and thrombus formation, the long-term consequences of which are unknown [24]. As the first-generation studies were conducted in 2009, the longest follow-up data thus far spans only 6 years. The unknown long-term impact of catheter-based RDN therapy upon renovascular integrity mandates future surveillance.

Although there are more studies in RDN compared to the other device-based hypertension therapies, most studies of RDN were limited by insufficient sample sizes, unregulated in-trial changes in medications, infrequent use of sham control, lack of appropriate blinding, unknown procedural success and limited involvement of hypertension specialists [25]. These inadequacies will be addressed in future trials of RDN following consensus amongst international experts [3•, 26].

### Future Directions

It is worth recalling that the technical approaches to RDN are constantly in evolution. It is likely that different iterations of the technology will be associated with different technical success and complication rates. There is an increasing utilisation of multi-electrode catheters for RF ablation and irrigated balloon catheters for US ablation [8]. Alternatively, catheters are being developed to introduce microinjection of neurotoxin (e.g. alcohol) to chemically ablate renal nerves, which has the theoretical advantage of facilitating deeper nerve injury whilst avoiding damage to the endothelium and intimal layers [27]. There is also a drive towards non-vascular techniques to achieve RDN. An alternative method of delivering RDN, via a transurethral approach to ablate the richly innervated renal pelvis, is now available for patients with bleeding disorders or renal artery anatomy that is unsuitable for current endovascular ablation catheters [28]. A wholly non-invasive system is also being developed by Kona Medical (Surround Sound™) which delivers focused ultrasound targeting the distal renal artery and bifurcation using advanced Doppler imaging. This system is currently being evaluated in a double blind sham-controlled clinical trial for patients with RHTN, with an expected completion date in March 2018 (clinicaltrials.gov: NCT02029885).

## Baroreflex Activation Therapy

### Mechanism of Action

Baroreflex activation therapy (BAT) is predicated upon the role of arterial baroreceptors in detecting carotid sinus and aortic arch distension in response to rises in arterial BP during systole, which then reflexively sends afferent nerve impulses into the nucleus tractus solitarius in the central nervous system. This in turn decreases the efferent sympathetic nervous system (SNS) discharge to the heart, peripheral vasculature and kidneys, resulting in negative inotropy, vasodilatation and reduced renin secretion, respectively. This also results in increased parasympathetic outflow with associated reduction in heart rate [29, 30]. Although the initial studies were conducted with bilateral placement of electrodes, the current iteration of this technology utilises a unilateral unipolar electrode which is surgically attached to the right carotid sinus, with the implantable pulse generator positioned subcutaneously in the subclavian area. The hardware and surgical technique has progressively evolved so that it is now feasible to be implanted under conscious sedation.

### Current Evidence

The first-generation Rheos™ device (requiring bilateral electrode placement) was initially evaluated in a feasibility study. In the non-randomised DEBuT-HT open-label trial with no comparator arm, implantation of the device in 45 patients with RHTN resulted in an average BP reduction of 21/12 mmHg at 3 months and 33/22 mmHg at 2 years [31]. In a separate small open-label study of 21 patients with RHTN there was a reduction of office BP by 31/14 mmHg and heart rate by 5 beats/min following BAT with the Rheos device [32]. This led to the subsequent Rheos Pivotal Trial: 265 patients were randomised in a 2:1 fashion to early (1 month post-implantation) or delayed (6 months post-implantation) device activation. The trial did not meet the endpoints for acute responders or procedural safety. Although there was no significant difference in the primary efficacy end point of ≥10 mmHg drop in systolic BP after 6-month follow-up, 42 % of participants in the early group vs 24 % of the delayed group achieved systolic BP <140 mmHg. Notably, the pivotal study also reported patients developing transient (4.4 %) or permanent (4.8 %) facial nerve injury [33]. Longer-term follow-up of this study reported that systolic BP reductions of 30 mmHg were sustained at 53 months [34]. A recent subgroup analysis of this trial indicated that unilateral therapy was more effective than bilateral therapy, and further sub-stratification suggests that right-sided stimulation may be more effective than left [35]. This may have important implications for future studies and clinical practice, as implanting a single lead could result in less procedure-related adverse events.

The next-generation update of this technology, Barostim neo™ system, utilises a smaller longer lasting generator and single lead carotid sinus stimulation [36]. A preliminary study in 33 patients with RHTN demonstrated BP reductions of 26/12 mmHg at 6 months with baseline systolic BP ≥160 mmHg. Importantly, the new technology allowed for shorter implantation and hospitalisation times, with less immediate procedure-related complications compared to the first generation system and no reports of either temporary or permanent facial nerve injury [37•].

BAT also appeared to be effective in the setting of chronic kidney disease (eGFR <90 mL/min/1.73 m^2^ or proteinuria). After 6 months, 23 patients managed with the Barostim neo™ system demonstrated a 17/9 mmHg fall in BP as compared to the 1/1 mmHg fall in the 21 patients in the control group (standard medical management), and a lower heart rate (−5 beats/min) [38].

### Future Directions

The Barostim Hypertension Pivotal Trial (clinicaltrials.gov: NCT01679132) is currently in progress and aims to enrol 310 patients with RHTN randomised to receiving optimal medical management alone or in combination with the baroreflex activation therapy. BAT may also have a role outside of BP management and is currently being evaluated as an adjunctive therapy in heart failure [39].

## Carotid Body Ablation

### Mechanism of Action

Carotid bodies are peripheral chemoreceptors that regulate sympathetic tone and respiratory minute ventilation in response to stimuli such as hypoxia, hypercapnia, hypoglycaemia, and acidosis. The ablation of carotid body (CB) function has been proposed as a target for circulatory regulation as increased efferent signalling from CBs is associated with hypertension that is reversible when the signalling is downregulated [40].

### Current Evidence

In COPD patients undergoing bilateral CB resection, systolic BP was reduced by 40 mmHg at 6 months post-operatively in a hypertensive sub-group, despite no long-term improvement in ventilatory parameters [41]. A recent proof of concept study of unilateral CB ablation as therapy for RHTN has demonstrated sustained office BP reduction of 23/12 mmHg at 6 months post-operatively in 8 out of 15 patients who had evidence of increased baseline CB activity. There were no serious adverse events reported, and hypoxic ventilatory drive was maintained [42].

### Future Directions

An ongoing uncontrolled observational study will assess the feasibility of unilateral endovascular CB ablation in a larger cohort of patients with RHTN. The targeted trial enrolment is set at 50 patients and is expected to report its findings in early 2017 (clinicaltrials.gov: NCT02099851). A separate trial in patients with RHTN aims to assess the effect of CB de-afferentation achieved by local US-guided infiltration of lidocaine followed by electrical stimulation (clinicaltrials.gov: NCT02519868). No prospective sham-controlled clinical trials have been registered to date.

## Central Iliac Arterio-Venous Anastomosis

### Mechanism of Action

In contrast to the above devices that aim to be sympathomodulatory, a central iliac arterio-venous (AV) anastomosis intends to reduce effective arterial volume, systemic vascular resistance (SVR) and cardiac afterload, thus lowering BP. This is achieved by creating a 4-mm fixed calibre conduit between the proximal arterial and low resistance venous circulation, typically the external iliac artery and vein, using a nitinol stent-like device (ROX AV coupler) placed under fluoroscopic guidance [43]. This diverts a calibrated amount of arterial blood (0.8 to 1.0 L/min) into the proximal large capacitance venous circuit, helping restore the Windkessel function of the central circulation, which may be of particular benefit in patients with greatly reduced vascular compliance due to arterial stiffening. The opening of the anastomosis results in an immediate and significant reduction of SVR and BP. The immediacy of the BP improvement suggests a negligible contribution from placebo/Hawthorne effects. It should be noted that whilst the primary physiological mechanism may be related to reduction in effective arterial volume, the device may be sympathomodulatory through increasing venous oxygenation and increasing right heart stretch through increased pre-load [44, 45].

### Current Evidence

Formation of a central iliac AV anastomosis was initially studied in patients with COPD with the intention of improving exercise capacity [46]. Subsequently, in an open-label study of 24 patients with COPD and mild hypertension, central iliac AV anastomosis was associated with an improvement in oxygen delivery and mixed venous oxygen saturations. There was an observed reduction in office BP from 145/86 to 132/67 mmHg at 12 months, without changes in medications [47].

The first investigation into central AV anastomoses in hypertension was the randomised controlled, open-label, ROX CONTROL HTN trial [48••], in which 83 patients were randomised to either standard care or insertion of AV coupler in addition to standard care. At 6 months, office BP and ambulatory BP were reduced by 27/20 and 14/14 mmHg, respectively, in the coupler group whilst no significant changes were observed in the control group. There was also a reduction in hospitalisations for hypertensive urgencies in the coupler group. Whilst there were no significant differences in the use of medications at baseline between the groups, in the coupler group 25 % of patients decreased their antihypertensives whilst 30 % of the control group had an increase in medications. Modified intention to treat analysis of the BP changes may have masked the true extent of coupler-induced BP reduction [48••].

The main complication reported in the coupler group was a 29 % incidence of ipsilateral venous stenosis which was successfully managed by venoplasty and/or stenting. In a small subset of patients who had failed to respond to prior RDN, central AV anastomosis resulted in significant reduction in both the office BP (34/22 mmHg) and ambulatory BP (12/15 mmHg). This may suggest that AV coupler therapy may be beneficial in cases where sympathomodulation has failed [49].

### Future Directions

Early clinical experience with the coupler suggest that this novel approach which addresses mechanical aspects of the circulation has promise, but more long-term safety and efficacy data are clearly required. Requirement for an appropriate sham-controlled study may be confounded by spontaneous patient reporting of a palpable thrill at the site of the AV anastomosis. Presently, ongoing evaluation of the therapy is taking place within a global registry study (clinicaltrials.gov: NCT1885390) and a US pivotal study is in the pipeline.

## Deep Brain Stimulation

### Mechanism of Action

The role of specific sub-structures within the brain in modulating autonomic activity for cardiovascular reflexes has previously been indicated in animal studies. The stimulation of the periaqueductal gray region of cats and rats has been shown to be linked to changes in blood pressure, heart rate and vasodilatation [50, 51].

### Current Evidence

To date, there is minimal data regarding the use of deep brain stimulation (DBS) as a treatment for hypertension. An initial description of DBS demonstrating benefit in refractory hypertension was based on targeted stimulation of the venterolateral periaquductal grey/periventricular grey for analgesia in stroke-associated hemibody central pain syndrome. The observed reduction in blood pressure of up to 33/13 mmHg appears to be independent of any analgesic effects, as it persisted even when pain levels returned to pre-surgical levels after several months [52]. The suggested mechanism may be via vasodilatation and reducing total peripheral resistance [53]. In a larger cohort of patients, utilizing DBS for chronic neuropathic pain or Parkinson’s disease resulted in improved vasomotor baroreflex sensitivity, decreased muscle sympathetic nerve activity and reduction in BP [54]. In common with other device therapies for hypertension, there also appears to be a range of BP responses to the therapy, where some could even be regarded as non-responders.

### Future Directions

Thus far, there are no registered trials of DBS as a device-based therapy for hypertension, although BP responses to DBS are being evaluated in an on-going study wherein the primary indication is for relief of chronic neuropathic pain in patients with spinal cord injury (clinicaltrials.gov: NCT02006433).

## Median Nerve Stimulator

### Mechanism of Action

A novel implantable non-constant neurostimulator has been designed by Valencia Technologies (eCoin) to be small enough, approximately 2 cm in diameter, to be placed subcutaneously overlying the median nerve as a potential therapy for RHTN. Median nerve stimulation, albeit by a different electroacupuncture system, is already supported by limited data. In anaesthetised male rats, delivery of electroacupuncture at points overlying the median nerve reduced the sympathoexcitatory BP response to gastric distension [55]. This BP-lowering effect of electroacupuncture over the median and deep peroneal nerves was also demonstrated in medicine-naive hypertensive patients, compared with a “placebo” electroaccupuncture group [56].

### Current Evidence

Although there are yet to be any peer-reviewed data for median nerve stimulation as a device-based therapy for RHTN, Valencia Technologies has indicated that their unpublished interim results showed the treatment group having a net change in systolic BP of 16.7 mmHg lower than that of the sham (implanted device but not activated) group (http://valenciatechnologies.com/first-application).

### Future Directions

An ongoing clinical trial aims to recruit 102 patients with RHTN into two trial arms, using a sham group as control (acntr.org.au:ACTRN12613000360718).

## Vagal Nerve Stimulation

### Mechanism of Action

Whilst there have been significant efforts in creating devices that modulate the SNS at various points, the parasympathetic nervous system has been largely neglected. The vagus nerve, together with the thoracic ganglia, is the principle source of parasympathetic innervation of the heart with resultant negative inotropic and chronotropic effects.

Vagal nerve stimulation (VNS) in Dahl salt-sensitive rats attenuated salt-induced hypertension and arrhythmias as compared to rats that had sham surgery [57]. On its own, VNS was able to reduce BP without inducing bradypnoea or bradycardia, which may be anticipated as a side effect of vagal stimulation [58], but stimulation-induced apnoea was observed following selective VNS in combination with beta-blockers [59]. Notably, the BP-lowering effect of VNS is partially additive to the effects of intravenous metoprolol [59] or enalapril [60].

### Current Evidence

Limited experience of the impact of VNS on blood pressure comes from a report of two patients undergoing carotid endarterectomy and coronary bypass surgery. Stimulating the vagus nerve resulted in a current- and frequency-dependent lowering of systolic BP and heart rate. It is important to recognise that episodes of atrio-ventricular block and ventricular asystole were observed at higher current and frequency, but were reversible upon termination of VNS [61].

### Future Directions

A study of selective VNS in patients undergoing carotid endarterectomy and coronary artery bypass grafting, with four patients enrolled, has been completed, but the results have not yet been published (clinicaltrials.gov: NCT00983632).

## Can Focused Patient Selection Improve Outcomes When Using Device Therapies of Hypertension?

Variations in responses to antihypertensive drug therapy across the population have been well described. It would be reasonable to hypothesise that the device-based therapies discussed here, each targeting unique pathophysiological pathways, would have their respective determinants of responders and non-responders. It has been long established that renal norepinephrine spillover is negatively correlated with age [62], suggesting that sympathomodulatory devices may be more efficacious in the younger adult, whereas devices that targets mechanisms other than sympathetic drive may be better suited to treating hypertension in the older adult.

The heterogeneity of BP responses to RDN suggests that it might be effective in a select group of patients, as subgroup analyses suggest a better response in non-African Americans as well as in younger patients and those with preserved renal function [63•]. This supports the hypothesis that younger patients with higher sympathetic tone and preserved vascular compliance may respond better to RDN. Conversely patients with isolated systolic hypertension and the associated structural hypertension may not be optimal candidates for RDN [64]. This interpretation may also extend to other sympathomodulatory aproaches such as BAT and CB ablation, but data is limited with these procedures. It has already been noted that assessment of baseline CB activity may be useful for selecting optimal responders to CB ablation [42]. Conversely, it may it might be hypothesised that a technique that is based on reducing effective arterial volume such as central AV anastomosis may be more appropriate in hypertensive patients with reduced vascular compliance and less dominated by elevated sympathetic tone. There is minimal data concerning median nerve and vagal nerve stimulation, which limits our ability to identify a subgroup of patients that may be responders to these technologies. Tests that would allow to identify patients with high likelihood of response to various device-based therapies are urgently needed as they will help to prevent patients from risks related to unnecessary procedures.

## Conclusions

Here, we have discussed seven different device-based therapies for hypertension (summarised in Table 1), with limited evidence of efficacy. Additionally, long-term data, including that of procedure- and device-related adverse events, is still to be determined. A consistent and ongoing challenge is the design and execution of rigorous, appropriately controlled studies with blinded endpoints. With that caveat, these technologies should all still be considered to be experimental therapeutic options for which there are insufficient data to presently support their use in routine clinical practice. In an ideal future, given the high costs of these technologies and the fact that there may be need for multiple treatments/battery replacement for some devices, there should be an algorithm to allow individualised treatment selection best suited for the patient’s hypertension phenotype. Furthermore, whilst device-based therapy is currently being explored predominantly for RHTN, it may be increasingly relevant to patients with multiple medications intolerances or those who simply wish to avoid long-term drug therapy.

**Table 1:** Summary of seven device-based therapies for hypertension

	Strength of evidence	Sham trial	Verifiable technical success	Reversible	Current estimated cost	Patient selection	Specific advantage	Potential adverse events
Renal denervation	Large sham-controlled RCT, no clear effect but potential confounders. Supportive registry data.	Yes	No	No	∼ €5–6000	Younger patients, non-African Americans, preserved renal function	Multiple approaches to achieve denervation	Diffuse arterial injury (from endovascular approach) with thermal energy
Baroreflex activation therapy	Large RCT for first-generation device completed. Ongoing large RCT (optimal medical therapy as control) for new-generation device.	Not yet	Yes	Yes. All electrical neuromodulation is reversible by switching off	∼€24,000 per device and ∼€15,000 for replacement battery every three years	Not enough experience	Potentially titratable activation with future iterations of device.	Potential nerve damage, although risk thought to be reduced with smaller, single-lead next-generation device. Battery replacement needed at 3–5-year intervals, generator failure possible. Open loop system—cannot titrate therapy to ambient BP levels
Carotid body ablation	Ongoing observational feasibility study	Not yet	Yes (surgical resection)	No	Not enough experience	Patients with high carotid body tone, although difficult to measure	Verifiable surgical resection	Not enough experience
Central iliac arterio-venous anastamosis	Small RCT (standard medical care as control) completed. Ongoing global registry study	Not easily achieved	Yes	Yes	∼ €4,000 for coupler (not including cost of procedure)	Nearly all patients respond but may best suit older patients with arterial stiffness/isolated systolic hypertension	Verifiable procedural success with immediate BP reduction. Targets mechanical aspects of the circulation rather than sympathetic drive	30 % incidence of ipsilateral venous stenosis, increased cardiac output
Deep brain stimulation	No registered trials, only observational data from non-blood pressure indications available	Not yet	Yes	Yes. All electrical neuromodulation is reversible by switching off	∼€40,000 per device (based on epilepsy therapy), uncertain costs for replacement battery	Not enough experience	Not enough experience	Not enough experience. Generator failure possible and battery replacement needed every 3–5 years. Open loop system - cannot titrate therapy to ambient BP levels
Median nerve stimulation	Ongoing sham-controlled RCT	In progress	Not enough experience	Yes. All electrical neuromodulation is reversible by switching off	Proposed to be low (<€2000)	Not enough experience	Minimally invasive procedure, inexpensive. Potentially titratable activation with future iterations of device.	Not enough experience. Battery replacement needed every 3–5 years. Open loop system—cannot titrate therapy to ambient BP levels
Vagal nerve stimulation	Animal model data and human case report available	Not yet	Not enough experience	Yes. All electrical neuromodulation is reversible by switching off	∼€20,000 (based on epilepsy therapy), uncertain for replacement battery	Not enough experience	Acts on parasympathetic system. Frequency- and current-dependent—facilitates titratable stimulation with future devices.	Battery replacement needed every 3–5 years. Open loop system—cannot titrate therapy to ambient BP levels

*RCT* randomised controlled trial

## Abbreviations

AV: Arteriovenous
BAT: Baroreflex activation therapy
BP: Blood pressure
CB: Carotid body
COPD: Chronic obstructive pulmonary disease
DBS: Deep brain stimulation
eGFR: Estimated glomerular filtration rate
RCT: Randomised controlled trial
RF: Radiofrequency
RHTN: Resistant hypertension
SNS: Sympathetic nervous system
SSAHT: Standardised stepped-care antihypertensive treatment
SVR: Systemic vascular resistance
US: Ultrasound
VNS: Vagus nerve stimulation
